# RETRACTION: A Bibliometric Analysis of Global Research on Microbial Immune Microenvironment in Melanoma From 2012 to 2022

**DOI:** 10.1111/srt.70165

**Published:** 2025-03-27

L. Sun, J. Ying, R. Guo, L. Jia, and H. Zhang, “A Bibliometric Analysis of Global Research on Microbial Immune Microenvironment in Melanoma From 2012 to 2022,” *Skin Research and Technology* 30, no. 8 (2024): e70017, https://doi.org/10.1111/srt.70017.

The above article, published online on 21 August 2024 in Wiley Online Library (wileyonlinelibrary.com), has been retracted by agreement between *Skin Research and Technology*; and John Wiley & Sons Ltd. After the article's publication, the journal became aware that the results of the bibliometric analysis could not be independently reproduced with the search query provided in the article. Upon request, the authors provided the list of articles object of study, which was found to contain significantly fewer articles than reported in the paper. Additionally, several of these articles were found not to meet the study's inclusion criteria. Accordingly, the article is retracted due to the unreliability of its findings. The authors have been informed of this decision.

